# Computed Tomography Patterns of *Pneumocystis jirovecii* Pneumonia According to Immune Status

**DOI:** 10.3390/diagnostics16111593

**Published:** 2026-05-22

**Authors:** Raúl Parra-Fariñas, Javier Infante-Armisen, Pilar Cifrián-Casuso, Moncef Belhassen-García, Javier Pardo-Lledías, José Antonio Parra-Blanco

**Affiliations:** 1 Service of Infectious Diseases, Hospital Universitario Marqués de Valdecilla, 39008 Santander, Spain; raul.parra@scsalud.es; 2 CIBERINFEC—Centro de Investigación Biomédica en Red de Enfermedades Infecciosas, Instituto de Salud Carlos III, 28029 Madrid, Spain; 3 Instituto de Investigación Marqués de Valdecilla (IDIVAL), 39008 Santander, Spain; pilar.cifrian@scsalud.es (P.C.-C.); javier.pardo@scsalud.es (J.P.-L.); 4 Department of Medical and Surgical Sciences, University of Cantabria, 39011 Santander, Spain; javier10infante@gmail.com; 5 Department of Radiology, Hospital Universitario Marqués de Valdecilla, 39008 Santander, Spain; 6 Infectious Diseases Unit, Internal Medicine Department, Hospital Universitario de Salamanca, 37007 Salamanca, Spain; mbelhassen@hotmail.com; 7 IBSAL—Instituto de Investigación Biomédica de Salamanca, CIETUS—Centro de Investigación de Enfermedades Tropicales, University of Salamanca, 37007 Salamanca, Spain; 8 Internal Medicine Department, Hospital Universitario Marqués de Valdecilla, 39008 Santander, Spain

**Keywords:** *Pneumocystis jirovecii* pneumonia, computed tomography, chest radiography, immunosuppression, opportunistic infections

## Abstract

**Background**: *Pneumocystis jirovecii* pneumonia (PJP) increasingly affects non-HIV immunocompromised patients; however, the spectrum of computed tomography (CT) findings in this population remains poorly defined. **Objectives**: To describe and compare chest CT findings of PJP in patients with and without HIV infection and to evaluate the impact of respiratory coinfections on imaging patterns. **Methods:** This retrospective single-centre cohort study included 72 adult patients with confirmed PJP diagnosed between 2011 and 2024, 27 HIV-positive and 45 non-HIV immunocompromised patients. Chest radiography was available in 71 patients and chest CT in 62. Imaging studies were independently reviewed for predefined patterns, including ground-glass opacities, alveolo-interstitial pattern, mosaic attenuation, crazy paving, pulmonary cysts, consolidation, and pleural effusion. CT findings were compared between HIV-positive and non-HIV patients, and a subgroup analysis was performed in non-HIV patients according to the underlying type of immunosuppression. Respiratory coinfections were recorded and classified based on microbiological results. **Results**: Chest radiography was normal in 32.4% of patients. An interstitial pattern tended to be more frequent in HIV-positive patients, whereas consolidations were more commonly observed in non-HIV patients (*p* = 0.051). On CT, ground-glass opacities were the predominant finding in both groups. HIV-positive patients more frequently demostrated an alveolo-interstitial pattern, mosaic attenuation, and pulmonary cysts, while consolidations and pleural effusions were more common in non-HIV patients, particularly among solid organ transplant recipients. Respiratory coinfections were identified in 63.9% of patients; however, no statistically significant differences in CT patterns were observed between patients with and without coinfections. **Conclusions**: PJP demonstrates different CT presentations according to immune status. HIV-positive patients more frequently demonstrated alveolo-interstitial patterns, mosaic attenuation, and pulmonary cysts, whereas consolidations were more commonly observed in non-HIV immunocompromised patients. Respiratory coinfections do not appear to significantly influence CT patterns.

## 1. Introduction

*Pneumocystis jirovecii* pneumonia (PJP) is a potentially life-threatening opportunistic infection that primarily affects immunocompromised patients [1]. Although historically considered an AIDS-defining illness, the incidence of HIV associated PJP has markedly decreased following the widespread use of trimethoprim–sulfamethoxazole prophylaxis and combination antiretroviral therapy [1,2]. In contrast, over recent decades, an increasing proportion of PJP cases has been reported in non-HIV immunocompromised patients, particularly those with hematological malignancies, solid organ or hematopoietic stem cell transplantation, autoimmune diseases, and exposure to immunosuppressive therapies [1,2,3,4].

Clinically, PJP typically presents with non-specific respiratory symptoms, including fever, non-productive cough, and progressive dyspnea and hypoxemia [3]. However, important differences have been described according to immune status. HIV-associated PJP usually follows a more indolent course, whereas non-HIV immunocompromised patients often present with a more acute and rapidly progressive disease, with higher inflammatory burden and worse outcomes [3,5,6].

These clinical differences are partly explained by distinct pathophysiological mechanisms. *Pneumocystis jirovecii* shows a marked tropism for the lung, where it disrupts alveolar function and gas exchange, leading to diffuse alveolar damage [4,7]. In non-HIV patients, a more intense host immune response is typically observed, resulting in greater inflammatory lung injury compared with HIV-infected patients [7,8,9].

From a radiological perspective, chest radiography has limited sensitivity in early PJP and may be normal despite clinically significant disease [10]. Computed tomography (CT), particularly high-resolution CT, plays a central role in the diagnostic evaluation of suspected PJP by allowing early detection and detailed characterization of parenchymal abnormalities [11]. While CT findings of PJP in HIV-positive patients are relatively well established, imaging manifestations in non-HIV immunocompromised patients appear more heterogeneous, with reported differences in the prevalence of consolidation, alveolo-interstitial patterns, mosaic attenuation, and crazy-paving [9,12,13,14].

Given the growing burden of PJP among non-HIV immunocompromised patients and the variability of reported imaging patterns, further characterization of CT findings in this population is warranted. In addition, limited data are available regarding whether imaging features vary according to the underlying type of immunosuppression and potential influence of coinfections [15,16]. The aim of this study was therefore to analyze and compare chest radiography and CT findings of PJP in patients with and without HIV infection, with particular emphasis on the non-HIV population, and to explore the potential influence of respiratory coinfections on CT patterns.

## 2. Material and Methods

### 2.1. Study Design and Population

A retrospective observational single-center study was conducted at a tertiary care hospital, including adult patients (≥18 years) with confirmed PJP diagnosed between 1 August 2011 and 31 December 2024. Patients were identified through institutional microbiology and clinical databases.

Patients were classified into two groups according to immune status: HIV-positive patients and non-HIV immunocompromised patients. The non-HIV group included individuals with hematological or solid malignancies, solid organ or bone marrow transplantation, autoimmune diseases, or prolonged exposure to systemic corticosteroids or other immunosuppressive therapies.

### 2.2. Clinical and Laboratory Data

Demographic and clinical data were extracted from electronic medical records, including age, sex, underlying immunosuppressive conditions, HIV status, CD4+ T-cell counts when available, and the presence of respiratory coinfections. Time intervals from symptom onset to hospital admission, diagnostic sampling, chest radiography, and computed tomography (CT) were recorded.

### 2.3. Diagnosis of Pneumocystis jirovecii Pneumonia

Given the absence of a single definitive reference standard for PJP, diagnosis was established based on the combination of compatible clinical and radiologic findings together with microbiological detection in respiratory samples.

Microbiological detection of *Pneumocystis jirovecii* was performed including bronchoalveolar lavage fluid, induced sputum, or lung biopsy, using conventional staining techniques and/or polymerase chain reaction (PCR) assays.

A positive PCR result alone was not considered sufficient for case inclusion. Only patients with compatible clinical manifestations and radiologic abnormalities were classified as confirmed PJP cases. This combined clinical–radiologic–microbiological definition was applied to reduce the risk of misclassifying colonization as active infection. All included patients received specific anti-PJP therapy according to the treating physician’s clinical judgment.

In accordance with international recommendations for the diagnosis of Pneumocystis pneumonia in immunocompromised patients [17], cases based solely on serum biomarkers without microbiological confirmation in respiratory samples were excluded.

### 2.4. Chest Radiography

Chest radiographs performed at hospital admission were reviewed. Projection type (anteroposterior, posteroanterior, or lateral), image quality, and the time interval from symptom onset to imaging were recorded.

Radiographic findings were classified as normal or abnormal. Abnormal findings were further categorized into interstitial, alveolo-interstitial, or consolidation patterns. Distribution was assessed according to lung zones (upper, middle, and lower), laterality (unilateral or bilateral), and intrapulmonary distribution (central, peripheral, or diffuse).

### 2.5. Computed Tomography

Chest CT examinations performed during the diagnostic work-up were analyzed. Both high-resolution CT (HRCT) and conventional CT studies were included. Slice thickness, the use of intravenous iodinated contrast, image quality, and timing relative to symptom onset and chest radiography were recorded.

CT images were evaluated for the presence of ground-glass opacities, alveolo-interstitial pattern, consolidation, micronodules, mosaic attenuation, pulmonary cysts, crazy-paving pattern, mediastinal lymphadenopathy, pleural effusion, pneumothorax, fibrosis, and bronchiectasis. The distribution of abnormalities was assessed according to lung zones, laterality, and intrapulmonary localization.

### 2.6. Image Analysis

All chest radiographs and CT examinations were independently reviewed by two radiologists (JAPB, PCC) with experience in thoracic imaging, who were blinded to clinical data except for the diagnosis of PJP. Discrepancies were resolved by consensus.

### 2.7. Respiratory Coinfections

Respiratory coinfections were defined as the identification of bacterial, viral, fungal, or parasitic pathogens in respiratory samples obtained at or near the time of PJP diagnosis. Coinfections were categorized according to pathogen type and the number of microorganisms identified.

### 2.8. Statistical Analysis

Continuous variables were expressed as mean ± standard deviation (SD) and compared using the Student’s *t* test or the Mann–Whitney *U* test, as appropriate. Categorical variables were expressed as absolute numbers and percentages and compared using the chi-square test or Fisher’s exact test. All statistical tests were two-sided, and a *p* value < 0.05 was considered statistically significant. Statistical analyses were performed using SPSS Statistics (versions 26–29; IBM Corp., Armonk, NY, USA).

### 2.9. Ethics Approval

This study was observational and non-interventional and did not involve additional diagnostic or therapeutic procedures beyond routine clinical care. All procedures were conducted in accordance with the Declaration of Helsinki and applicable data protection regulations. The study was approved by the Ethics Committee for Research on Medicines and Medical Devices of Cantabria (CEIM; internal code 2023.431; approval date: 15 December 2023). Due to the retrospective design and the use of anonymized data, the requirement for informed consent was waived in accordance with national regulations and institutional policies.

## 3. Results

### 3.1. Patient Characteristics

A total of 72 patients with confirmed PJP were included in the study, of whom 27 (37.5%) were HIV-positive and 45 (62.5%) were non-HIV immunocompromised. The overall mean age was 58.7 years, and 51 patients (70.8%) were male.

HIV positive patients were significantly younger than non-HIV patients (49.3 vs. 64.4 years, *p* = 0.004) and were more frequently male (88.9% vs. 60.0%, *p* = 0.009). In the HIV-positive group, most HIV-positive patients had CD4+ T-cell counts below 200 cells/µL, with a mean CD4+ count of 51.2 ± 46.9 cells/µL. In the non-HIV group, the most common causes of immunosuppression were active malignancy and solid organ transplantation.

The time from symptom onset to hospital admission and to diagnostic sampling was significantly longer in HIV-positive patients compared with non-HIV patients (*p* < 0.01 for both). Baseline demographic and clinical characteristics are summarized in Table 1.

### 3.2. Chest Radiography Findings

Chest radiographs at admission were available for 71 patients (27 HIV-positive and 44 non-HIV). Projection type and image quality did not differ between groups. The mean interval from symptom onset to chest radiography was significantly longer in HIV positive patients than in non-HIV patients (20.3 vs. 8.8 days, *p* = 0.001). See Supplementary Table S1.

Chest radiographs were normal in 23 patients (32.4%), more frequently in the non-HIV group, although this difference was not statistically significant. Among patients with abnormal radiographs, an interstitial pattern was the most common finding in both groups.

In subgroup analysis, interstitial patterns tended to be more frequent in HIV-positive patients (81.0% vs. 51.9%), whereas consolidations were commonly observed in non-HIV patients (44.4% vs. 19.0%), although these differences did not reach statistical significance (*p* = 0.051). Consolidations were significantly more often unilateral in non-HIV patients compared with HIV-positive patients (83.3% vs. 9.1%, *p* = 0.020). No statistically significant differences were observed in lung zone involvement or intrapulmonary distribution. Detailed chest radiography findings are presented in Table 2.

### 3.3. Computed Tomography Findings

Chest CT examinations were available for 62 patients (20 HIV-positive and 42 non-HIV). CT acquisition parameters were comparable between groups with no significant differences in CT technique, slice thickness, intravenous contrast administration, or image quality. HRCT was performed in 54.8% of examinations, most CT studies were acquired with a slice thickness ≤1.25 mm (72.6%), and 61.3% were performed without intravenous contrast. Overall, image quality was rated as optimal in 95.2% of examinations. The mean interval from symptom onset to CT was significantly longer in HIV-positive patients than in non-HIV patients (25.3 vs. 13.0 days, *p* = 0.003). See Supplementary Table S2.

CT examinations were normal in seven patients (one HIV-positive and six non-HIV). Among patients with abnormal CT findings, ground-glass opacity was the most frequent pattern overall (61.8%). The distribution of CT patterns differed significantly between HIV-positive and non-HIV patients (*p* = 0.029).

In HIV-positive patients, ground-glass opacity was the most common finding, followed by alveolo-interstitial patterns. In contrast, in non-HIV patients, ground-glass opacity was followed by consolidations. Pulmonary cysts were observed exclusively in HIV-positive patients (26.3% vs. 0%, *p* = 0.001). Mosaic attenuation was significantly more frequent in HIV-positive patients (*p* = 0.034), whereas pleural effusions were more common in non-HIV patients (33.3% vs. 5.3%, *p* = 0.022). No significant differences were observed in lung zone distribution, laterality, or intrapulmonary localization. CT findings are detailed in Table 3.

### 3.4. Relationship Between Chest Radiography and CT

In most cases, CT was performed within five days after chest radiography. A substantial proportion of patients with normal chest radiographs demonstrated abnormal findings on CT. Conversely, several patients with consolidations on chest radiography showed predominantly interstitial or ground-glass patterns on CT. See Supplementary Table S3.

### 3.5. Imaging Patterns According to Type of Immunosuppression in Non-HIV Patients

Within the non-HIV group, transplant recipients represented a substantial proportion of patients, followed by patients with hematological malignancies and solid tumors (see Table 1). No significant differences were observed in chest radiography patterns among these subgroups.

**Figure 1:**
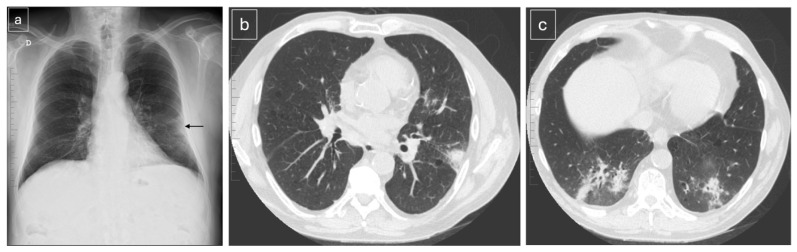
Chest imaging findings in an immunocompromised solid organ transplant recipient with a four-day history of fever. The initial chest radiograph (**a**) demonstrates a pleural-based consolidation in the left mid-lung zone (arrow). A follow-up chest CT performed five days later (**b**) confirms the presence of this consolidation and additionally reveals newly developed consolidations in both lower lobes (**c**). The diagnosis of *Pneumocystis jirovecii* pneumonia was subsequently confirmed by polymerase chain reaction (PCR) analysis of bronchoalveolar lavage fluid.

On CT, ground-glass opacity was the predominant pattern across all groups except in SOT recipients, in whom consolidations were the most frequent finding (Figure 1a–c). Alveolo-interstitial patterns were more frequent in patients with solid tumors and the crazy-paving pattern patients with hematological malignancies and solid tumors. These findings are summarized in Table 4.

### 3.6. Respiratory Coinfections

Overall, 46 patients (63.9%) had at least one respiratory coinfection; 19 (70.4%) in HIV positive and 27 (60%) in non-HIV patients. Viral (15/46) and bacterial pathogens (11/46) were the most frequently identified, and (18/46) had mixed infections involving two or more microorganisms. Coinfection rates did not differ significantly between HIV-positive and non-HIV patients. See Supplementary Table S4.

Respiratory coinfections were frequent but did not significantly modify the predominant chest radiography or CT patterns, either in the overall cohort or when stratified by HIV status. Coinfection data and corresponding imaging findings are summarized in Table 5.

## 4. Discussion

In this retrospective cohort of patients with confirmed PJP, we identified relevant differences in chest radiography and CT findings according to immune status. Although ground-glass opacity represented the predominant imaging feature in both groups, non-HIV immunocompromised patients more commonly presented with consolidations, unilateral involvement, and pleural effusions, whereas HIV-positive patients more frequently exhibited diffuse interstitial patterns, mosaic attenuation, and pulmonary cysts. These findings support the concept that PJP represents a heterogeneous radiological entity whose presentation is strongly influenced by the underlying type of immunosuppression.

The demographic and clinical differences observed between groups are consistent with previous studies. HIV-positive patients were younger and predominantly male, reflecting the epidemiology of HIV infection, while non-HIV patients were older and frequently had comorbidities related to malignancy, transplantation, or immunosuppressive therapies [3,6]. In addition, the longer interval from symptom onset to diagnosis observed in HIV-positive patients supports earlier observations that HIV-associated PJP often follows a more indolent clinical course, whereas non-HIV PJP tends to present acutely, leading to earlier imaging and diagnostic evaluation [4,14].

Chest radiography demonstrated limited sensitivity in both groups, with a substantial proportion of normal examinations at presentation, particularly among non-HIV patients [10]. This finding is in line with previous reports describing normal or subtle radiographic findings in early PJP and highlights the limited negative predictive value of chest radiography in immunocompromised patients with suspected infection. In our cohort, interstitial patterns predominated overall, but consolidations were more frequent and more often unilateral in non-HIV patients. This radiographic presentation may reflect a more intense inflammatory response and may overlap with other causes of pneumonia, potentially complicating the initial radiological assessment [9].

CT proved to be substantially more informative than chest radiography, revealing abnormalities in the majority of patients with normal radiographs. Ground-glass opacity was the most frequent CT finding, consistent with the known pathophysiology of PJP. However, the distribution of associated CT patterns differed according to immune status. HIV-positive patients more commonly demonstrated alveolo-interstitial involvement, mosaic attenuation, and pulmonary cysts, whereas non-HIV patients showed a higher prevalence of consolidations and pleural effusions. These differences likely reflect variations in host immune response and disease evolution, which are known to influence radiological presentation [5,18].

Pulmonary cysts were observed exclusively in HIV-positive patients, in agreement with previous studies reporting an association between cyst formation and chronic disease evolution in HIV-related PJP [18]. In contrast, pleural effusions were significantly more frequent in non-HIV patients. Although pleural involvement has traditionally been considered uncommon in PJP, several reports have described higher rates of pleural effusions in non-HIV populations, possibly related to concomitant conditions [5,15].

Our subgroup analysis within the non-HIV population further highlights the heterogeneity of PJP imaging manifestations according to the underlying immunosuppressive condition. Although ground-glass opacity remained the predominant CT finding, consolidations were more frequently observed among SOT recipients. recipients. Nevertheless, these subgroup findings should be interpreted with caution due to the limited number of patients included in each subgroup. Overall, our findings are consistent with previous studies reporting heterogeneous CT presentations of PJP in non-HIV immunocompromised patients [9,14,16,19].

Respiratory coinfections were frequent in our cohort; however, they did not significantly modify the predominant chest radiography or CT patterns. This observation suggests that, although coinfections may influence clinical severity and outcomes, the dominant imaging features of PJP are primarily driven by host immune status rather than by the presence of additional pathogens [16].

From a clinical and radiological perspective, our findings underscore the importance of early CT evaluation in immunocompromised patients with suspected PJP, particularly in non-HIV patients presenting with acute respiratory symptoms and inconclusive chest radiographs. Awareness of immune status-dependent CT phenotypes may facilitate earlier radiological suspicion and support timely diagnosis in this high-risk population.

This study has several limitations. First, its retrospective and single-centre design may limit the generalizability of the findings. In addition, the relatively small sample size, particularly within subgroup analyses according to the underlying immunosuppressive condition among non-HIV patients, may have limited the statistical power to detect more subtle differences in imaging patterns or to adequately evaluate the potential impact of respiratory coinfections. Consequently, these subgroup analyses should be interpreted as exploratory. Further multicenter studies including larger patient cohorts are warranted to confirm and validate these findings. Nevertheless, our results provide additional insight into the heterogeneous CT manifestations of PJP across different immunocompromised populations.

## 5. Conclusions

In conclusion, *Pneumocystis jirovecii* pneumonia demonstrates distinct radiological patterns according to immune status. Non-HIV immunocompromised patients more frequently present with consolidations, unilateral disease, and pleural effusions, whereas HIV-positive patients more commonly exhibit diffuse interstitial changes, mosaic attenuation, and pulmonary cysts. Recognition of these differences and early use of CT imaging are essential to optimize radiological diagnosis in immunocompromised patients with suspected PJP.

## Figures and Tables

**Table 1:** Demographic and clinical characteristics of HIV and non-HIV patients with PJP.

	Total n = 72 (%)	HIV n = 27 (%)	Non-HIV n = 45 (%)	*p* Value
**Age**. years (mean ± SD)	58.5 ± 13.6	49.3 ± 10.0	64.4 ± 12.3	0.004
**Male sex**	51 (70.8)	24 (88.9)	27 (60.0)	0.009
**Immunosuppression**				
*CD4+ T-cell count* < *200* cells/µL; (*mean* ± *SD*)		25 (96.2); (51.2 ± 46.9)		
*Hematologic malignancy*			10 (22.2)	
*HSCT*			5 (11.1)	
*SOT*			10 (22.2)	
*Solid tumor*			8 (17.8)	
Other *			12 (26.7)	
**Any coinfection**	46 (63.9)	19 (70.4)	27 (60.0)	0.506
Symptom onset to hospital admission (days) mean ± SD	11.87 ± 15.128	19.12 ± 14.9	7.69 ± 13.744	0.002
Symptom onset to sample collection (days) mean ± SD	18.51 ± 13.89	24.69 ± 13.04	14.93 ± 13.214	0.004

HIV: Human immunodeficiency virus; SD: standard deviation. HSCT: hematopoietic stem cell transplant; SOT: solid organ transplant. * Other included: 10 patients receiving chronic corticosteroids therapy, 2 patients with Cirrhosis, one receiving methotrexate and one receiving Adalimumab.

**Table 2:** Chest radiography findings at admission.

	Total n = 71 (%)	HIV n = 27 (%)	Non-HIV n = 44 (%)	*p* Value
**Normal**	23 (32.4)	6 (22.2)	17 (38.6)	0.242
**Abnormal**	48 (67.6)	21 (77.8)	27 (61.4)	
*Interstitial pattern*	31 (64.6)	17 (81.0)	14 (51.9)	0.69
*Consolidation*	16 (33.3)	4 (19.0)	12 (44.4)	
*Alveolo-interstitial*	1 (2.1)	0 (0.0)	1 (3.7)	
**Lung distribution**				
**Interstitial pattern**	31 (64.6)	17 (81.0)	14 (51.9)	0.075
Field distribution				1.000
*Upper*	2 (6.5)	1 (5.9)	1 (7.1)	
*Middle*	6 (19.4)	3 (17.6)	3 (21.4	
*Upper and middle*	3 (9.7)	2 (11.8)	1 (7.1)	
*Middle and lower*	6 (19.4)	3 (17.6)	3 (21.4)	
*Lower*	2 (6.5)	1 (5.9)	1 (7.1)	
*All lung zones*	12 (38.7)	7 (41.2)	5 (35.7)	
Unilateral/bilateral				0.18
*Unilateral*	10 (32.3)	2 (11.8)	8 (57.1)	
*Bilateral*	21 (67.7)	15 (71.4)	6 (42.9)	
Intrapulmonary distribution				0.502
*Central*	10 (32.3)	7 (41.2)	3 (21.4)	
*Peripheral*	5 (16.1)	2 (11.8)	3 (21.4)	
*Diffuse*	16 (51.6)	8 (47.1)	8 (57.1)	
**Consolidation**	16 (33.3)	4 (19.0)	12 (46.2)	0.051
Field distribution				0.430
*Upper*	3 (18.8)	0 (0.0)	3 (25.0)	
*Middle*	4 (25.0)	1 (25.0)	3 (25.0)	
*Upper and middle*	1 (6.2)	1 (25.0)	0 (0.0)	
*Lower*	6 (37.5)	2 (50)	4 (33.3)	
*All lung zones*	2 (12.5)	0 (0.0)	2 (16.7)	
Unilateral/bilateral				0.020
*Unilateral*	11 (68.8)	1 (25.0)	10 (83.3)	
*Bilateral*	5 (31.3)	3 (75.0)	2 (16.7)	
Intrapulmonary distribution				0.791
*Central*	3 (18.8)	1 (25.0)	2 (32.1)	
*Peripheral*	7 (43.8)	1 (25.0)	6 (50.0)	
*Diffuse*	6 (37.5)	2 (50.0)	4 (33.3)	
**Alveolo-interstitial**	1 (2.1)	0 (0.0)	1 (3.7)	
*All lung zones*	1 (100.0)	0 (0.0)	1 (100.0)	
*Bilateral*	1 (100.0)	0 (0.0)	1 (100.0)	
*Diffuse*	1 (100.0)	0 (0.0)	1 (100.0)	
**Pleural effusion**	2 (4.9)	0 (0.0)	2 (16.7)	0.232

HIV: Human immunodeficiency virus.

**Table 3:** Chest CT findings.

	Total n = 62 (%)	HIV n = 20 (%)	Non-HIV n = 42 (%)	*p* Value
**Normal**	7 (11.3)	1 (5.0)	6 (14.3)	0.687
**Abnormal**	55 (88.7)	19 (95.0)	36 (85.7)	
*Ground-glass opacity*	34 (61.8)	10 (52.6)	24 (66.7)	0.029
*Alveolo-interstitial*	11 (20.0)	7 (36.8)	4 (11.1)	
*Consolidation*	9 (16.4)	1 (5.3)	8 (22.2)	
*Micronodular*	1 (1.8)	1 (5.3)	0 (0.0)	
**Lung distribution**				
**Ground-glass opacity**	34 (63.6)	10 (57.9)	24 (66.7)	
Field distribution				0.912
*Upper*	2 (5.9)	0 (0.0)	2 (8.3)	
*Upper and middle*	15 (26.6)	4 (40.0)	11 (45.8)	
*Middle and lower*	4 (11.8)	1 (10.0)	3 (12.5)	
*Lower*	2 (5.9)	1 (10.0)	1 (4.2)	
*All lung zones*	11 (32.4	4 (40.0)	7 (29.2)	
Unilateral/bilateral				1.000
*Unilateral*	2 (5.9)	0 (0.0)	2 (8.3)	
*Bilateral*	32 (94.1)	10 (100.0)	22 (91.7)	
Intrapulmonary distribution				1.000
*Central*	1 (2.9)	0 (0.0)	1 (4.2)	
*Peripheral*	3 (8.8)	1 (10.0)	2 (8.3)	
*Diffuse*	30 (88.2)	9 (90.0)	21 (87.5)	
**Alveolo-interstitial pattern**	11 (20.0)	7 (36.8)	4 (11.1)	
Field distribution				0.716
*Upper and middle*	4 (36.4)	3 (42.9)	1 (25.0)	
*Middle and lower*	1 (9.1)	1 (14.3)	0 (0.0)	
*All lung zones*	6 (54.5)	3 (42.9)	3 (75.0)	
Unilateral/bilateral				
*Bilateral*	11 (100.0)	7 (100.0)	4 (100.0)	
Intrapulmonary distribution				
*Diffuse*	11 (100.0)	7 (100.0)	4 (100.0)	
**Consolidation**	9 (16.4)	1 (5.3)	8 (22.2)	
Field distribution				1.000
*Upper*	2 (22.2)	0 (0.0)	2 (25.0)	
*Upper and middle*	2 (22.2)	0 (0.0)	2 (25.0)	
*Middle and lower*	2 (22.2)	0 (0.0)	2 (25.0)	
*Lower*	2 (22.2)	1 (100.0)	1 (12.5)	
*All lung zones*	1 (11.1)	0 (0.0)	1 (12.5)	
Unilateral/bilateral				1.000
*Unilateral*	5 (55.6)	1 (100.0)	4 (50.0)	
*Bilateral*	4 (44.4)	0 (0.0)	4 (50.0)	
Intrapulmonary distribution				1.000
*Central*	1 (11.1)	0 (0.0)	1 (12.5)	
*Peripheral*	2 (22.2)	0 (0.0)	2 (25.0)	
*Diffuse*	6 (66.7)	1 (100.0)	5 (62.5)	
**Micronodular**	1 (2.4)	1 (9.1)	0 (0.0)	
Field distribution				
*All lung zones*	1 (100.0)	1 (100.0)	0 (0.0)	
Unilateral/bilateral				
*Bilateral*	1 (100.0)	1 (100.0)	0 (0.0)	
Intrapulmonary distribution				
*Diffuse*	1 (100.0)	1 (100.0)	0 (0.0)	
**Mosaic pattern**	24 (51.1)	12 (66.7)	12 (41.4)	0.034
**Pulmonary cysts**	5 (9.1)	5 (26.3)	0 (0.0)	0.001
**Crazy paving**	22 (43.6)	8 (42.1)	14 (38.9)	0.817
**Mediastinal lymphadenopathy**	3 (5.5)	2 (10.5)	1 (2.8)	0.272
**Pleural effusion**	13 (23.6)	1 (5.3)	12 (33.3)	0.022
*Unilateral*	5 (38.5)	0 (0.0)	5 (41.7)	
*Bilateral*	8 (61.5)	1 (100.0)	7 (58.3)	
**Pneumothorax**	2 (5.7)	0 (0.0)	2 (7.7)	0.539
**Fibrosis**	1 (2.8)	0 (0.0)	1 (4.2)	1.000
**Bronchiectasis**	9 (23.7)	3 (23.1)	6 (24.0)	1.000

HIV: Human immunodeficiency virus.

**Table 4:** Imaging patterns by type of immunosuppression in non-HIV patients.

	Hematologic Malignancy n = 10 (%)	HSCT n = 5 (%)	SOT n = 10 (%)	Solid Tumor n = 8 (%)	Other n = 12 (%)
**Chest radiography**	**n = 10 (100.0)**	**n = 5 (100.0)**	**n = 10 (100.0)**	**n = 8 (100.0)**	**n = 11 (91.7)**
*Normal*	5 (50.0)	2 (40.0)	4 (40.0)	1 (12.5)	5 (45.4)
*Interstitial pattern*	4 (40.0)	3 (60.0)	1 (10.0)	4 (50.0)	3 (27.3)
*Consolidation*	1 (10.0)	0 (0.0)	5 (50.0)	3 (37.5)	3 (27.3)
**CT**	**n = 9 (90.0)**	**n = 4 (80.0)**	**n = 10 (100.0)**	**n = 7 (100.0)**	**n = 12 (100.0)**
*Normal*	0 (0.0)	0 (0.0)	2 (20.0)	0 (0.0)	4 (33.3)
*Ground-glass opacity*	8 (89.0)	3 (75.0)	3 (30.0)	5 (71.4)	5 (41.7)
*Alveolo-interstitial*	1 (11.0)	0 (0.0)	0 (0.0)	2 (28.6)	1 (8.3)
*Consolidation*	0 (0.0)	1 (25.0)	5 (50.0)	0 (0.0)	2 (16.7)
*Mosaic attenuation*	3 (33.3)	2 (50.0)	0 (0.0)	3 (42.9)	4 (33.3)
*Crazy paving*	6 (66.7)	1 (25.0)	1 (20.0)	5 (71.4)	1 (8.3)
*Pleural effusion*	4 (44.4)	0 (0.0)	3 (30.0)	4 (57.1)	1 (8.3)

HSCT: hematopoietic stem cell transplant; SOT: solid organ transplant; CT: computed tomography.

**Table 5:** Radiography and CT findings in patients without and with coinfections.

	No Coinfection			Any Coinfection		
Chest Radiography	HIV n = 8 (%)	Non-HIV n = 18 (%)	*p* Value	VIH n = 19 (%)	Non-VIH n = 27 (%)	*p* Value
**Normal**	3 (37.5)	4 (22.2)	0.354	3 (15.8)	13 (48.1)	0.109
**Abnormal**	5 (62.5)	13 (76.5)		16 (84.2)	14 (51.9)	
*Interstitial pattern*	4 (80.0)	5 (38.5)	0.498	13 (81.2)	9 (64.3)	0.417
*Consolidation*	1 (20.0)	7 (53.8)		3 (18.8)	5 (35.7)	
*Alveolo-interstitial*	0 (0.0)	1 (7.7)		0 (0.0)	0 (0.0)	
**CT**	**HIV n = 5 (%)**	**Non-HIV n = 17 (%)**	***p* value**	**HIV n = 15 (%)**	**Non-HIV n = 25 (%)**	***p* value**
**Normal**	1 (20.0)	4 (23.5)		0 (0.0)	2 (8.0)	
**Abnormal**	4 (80.0)	13 (76.5)		15 (100.0)	23 (92.0)	
*Ground Glass opacity*	2 (50.0)	9 (69.2)	0.238	9 (60.0)	15 (65.2)	0.178
*Alveolo-interstitial*	2 (50.0)	1 (7.7)		5 (33.3)	3 (13.0)	
*Consolidation*	0 (0.0)	3 (23.1)		1 (6.7)	5 (21.7)	
**Cysts**	1 (25.0)	0 (0.0)	0.235	4 (26.7)	0 (0.0)	0.018
**Mosaic pattern**	3 (75.0)	7 (53.8)	0.452	9 (60.0)	5 (21.7)	0.017
**Crazy paving**	1 (25.0)	7 (53.8)	0.312	7 (46.7)	7 (30.4)	0.311

HIV: Human immunodeficiency virus.

## Data Availability

Data available from the corresponding author upon reasonable request.

## Supplementary Materials

The following supporting information can be downloaded at: https://www.mdpi.com/article/10.3390/diagnostics16111593/s1. Supplementary Table S1. Radiographic technique and time between symptom onset and Chest X-ray. Supplementary Table S2. CT technique and time between symptom onset and CT. Supplementary Table S3. Findings in chest radiography and CT in 61 patients with both techniques. Supplementary Table S4. Coinfections.
