# Supplementary material for: Computed Tomography Patterns of Pneumocystis jirovecii Pneumonia According to Immune Status
Source: Diagnostics (Basel). 2026 May 22;16(11):1593. doi: 10.3390/diagnostics16111593 (PMC13256207; doi:10.3390/diagnostics16111593)
Supplement: Supplementary file 1 [file diagnostics-16-01593-s001.zip › diagnostics-4242411-supplementary.pdf]

Supplementary Table S1. Radiographic technique and time between symptom onset and Chest X-ray

	Total n= 71 (%)	HIV n=27 (%)	Non-HIV n=44 (%)	*p* value
**Projection.**				
AP	30 (42.3)	11 (40.7)	19 (42.3)	1.000
PA	13 (18.3)	5 (18.5)	8 (18.2)	
PA y lateral	28 (39.4)	11 (40.7)	17 (38.6)	
**Image quality**				
Optimal	69 (97.2)	26 (96.3)	43 (97.7)	1.000
Suboptimal	2 (2.8)	1 (3.7)	1 (2.3)	
**Symptom onset to radiography (days) mean ± SD**	13.09 ± 14.861	20.31 ± 15.19	8.82 ± 13.03	0.001
**Hospital admission to radiography (days) mean ± SD**	1.17 ± 5.451	1.15 ± 5.082	1.18 ± 5.723	0.666

HIV: Human immunodeficiency virus; AP: anterior-posterior projection; PA: posterior-anterior projection; SD: standard deviation

Supplementary Table S2. CT technique and time between symptom onset and CT

	Total n=62 (%)	HIV n=20 (%)	Non-HIV n=42 (%)	*p* value
**CT**				
HRCT	34 (54.8)	12 (60.0)	22 (52.4)	0.433
Conventional	28 (45.2)	8 (40.0)	20 (47.6)	
**Endovenous contrast**				
No	38 (61.3)	13 (65.0)	25 (59.5)	0.679
Yes	24 (38.7)	7 (35.0)	17 (40.5)	
**Image quality**				
Optimal	59 (95.2)	20 (100.0)	39 (92.9)	0.545
Suboptimal	3 (4.8)	0 (0.0)	3 (7.1)	
**Symptom onset to CT (days) mean ± SD**	16.79 ± 15.21	25.26 ± 17.78	12.95 ± 12.135	0.003
**Hospital admission to CT (days) mean ± SD**	5.74 ± 8.0	5.10 ± 7.412	6.05 ± 8.334	0.666
**Chest X ray to CT (days) mean ± SD**	4.95 ± 6.791	4.85 ± 7.345	4.95 ± 6.599	0.999

CT: computed tomography; HRCT: High resolution computed tomography; SD: standard deviation

Supplementary Table S3. Findings in chest radiography and CT in 61 patients with both techniques

Chest radiography		CT			
**Pattern**	**Total n=61 (%)**	**Normal**	**Ground glass**	**Alveolo-interstitial**	**Consolidation**
**Normal**	17 (27.9)	5 (29.4)	8 (47.1)	1 (5.9)	3 (17.7)
**Interstitial**	29 (47.6)		21 (72.4)	7 (24.1)	1 (3.4)
**Consolidation:**	14 (23.0)	1 (7.1)	5 (35.7)	3 (21.4)	5 (35.7)
**Alveolo-interstitial**	1 (1.6)			1 (100.0)	

CT: computed tomography

Supplementary Table S4. Coinfections

Coinfections	Total n=72(%)	HIV n=27(%)	Non-HIV n=45(%)	*p* value
No	26 (36.1)	8 (29.6)	18 (40.0)	
Yes	46 (63.9)	19 (70.4)	27 (60.0)	0.506
Coinfection type				
Bacterial	11 (23.9)	4 (21.1)	7 (25.9)	
Viral	15 (32.6)	5 (26.3)	10 (37.0)	
Fungal	2 (4.3)	0 (0.0)	2 (7.4)	
Bacterial and viral	7 (15.2)	3 (15.8)	4 (14.8)	
Bacterial and fungal	2 (4.3)	2 (10.6)	0 (0.0)	
Viral and fungal	3 (6.5)	2 (10.6)	1 (3.7)	
Viral and parasitic	1 (2.2)	1 (5.3)	0 (0.0)	
Bacterial.viral and fungal	5 (10.9)	2 (10.6)	3 (11.1)	

HIV: Human immunodeficiency virus
